# Blood lactate as a predictor for in-hospital mortality in patients admitted acutely to hospital: a systematic review

**DOI:** 10.1186/1757-7241-19-74

**Published:** 2011-12-28

**Authors:** Ole Kruse, Niels Grunnet, Charlotte Barfod

**Affiliations:** 1 Faculty of Health Sciences, University of Copenhagen, Denmark; 2 Department of Biomedical Sciences, Faculty of Health Sciences, University of Copenhagen, Denmark; 3 Department of Anaesthesiology and Intensive Care, Hillerød Hospital, Denmark

**Keywords:** Lactate, triage, mortality, trauma, emergency department, intensive care unit, point-of-care

## Abstract

**Background:**

Using blood lactate monitoring for risk assessment in the critically ill patient remains controversial. Some of the discrepancy is due to uncertainty regarding the appropriate reference interval, and whether to perform a single lactate measurement as a screening method at admission to the hospital, or serial lactate measurements. Furthermore there is no consensus whether the sample should be drawn from arterial, peripheral venous, or capillary blood. The aim of this review was:

1) To examine whether blood lactate levels are predictive for in-hospital mortality in patients in the acute setting, i.e. patients assessed pre-hospitally, in the trauma centre, emergency department, or intensive care unit.

2) To examine the agreement between arterial, peripheral venous, and capillary blood lactate levels in patients in the acute setting.

**Methods:**

We performed a systematic search using PubMed, Cochrane Central Register of Controlled Trials, Cochrane Database of Systematic Reviews, and CINAHL up to April 2011. 66 articles were considered potentially relevant and evaluated in full text, of these ultimately 33 articles were selected.

**Results and Conclusion:**

The literature reviewed supported blood lactate monitoring as being useful for risk assessment in patients admitted acutely to hospital, and especially the trend, achieved by serial lactate sampling, is valuable in predicting in-hospital mortality. All patients with a lactate at admission above 2.5 mM should be closely monitored for signs of deterioration, but patients with even lower lactate levels should be considered for serial lactate monitoring. The correlation between lactate levels in arterial and venous blood was found to be acceptable, and venous sampling should therefore be encouraged, as the risk and inconvenience for this procedure is minimal for the patient. The relevance of lactate guided therapy has to be supported by more studies.

## Introduction

Handheld point-of-care testing (POCT) has made it possible to assess blood lactate easy and fast, but the use of blood lactate monitoring for risk assessment in the critically ill patient remains controversial. In the intensive care unit (ICU), blood lactate monitoring is widely used as an indirect marker of tissue hypoxia [1,2]. In the assessment of the traumatized patient [3] and the critically ill patient admitted to Emergency Department (ED) [2,4], some hospitals also include blood lactate monitoring. The different policies between hospitals and departments reflect the controversy regarding the usefulness of the blood sample. Some of the discrepancy is due to uncertainty regarding the appropriate reference interval, and whether to perform a single lactate measurement as a screening method at admission to the hospital, or serial lactate measurements. Furthermore there is some discrepancy whether the sample should be obtained from arterial, central venous, peripheral venous, or capillary blood. Previous reviews do not address the question of single versus serial sampling, cut off levels, or point of sampling [1,5-8].

The aim of this review was to appraise the scientific literature to uncover whether elevated blood lactate levels are predictive for in-hospital mortality in patients in the acute setting, i.e. patients assessed pre-hospitally, in the trauma center, ED, or ICU. Furthermore to examine the agreement between arterial, peripheral venous, and capillary lactate levels in patients in the acute setting.

### Biochemistry

**Figure 1:**
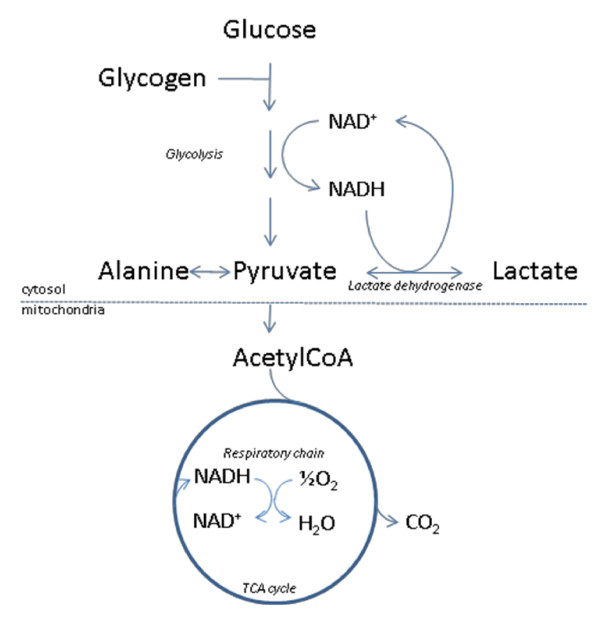
**Outline of lactate metabolism**. With insufficient oxygen supply, pyruvate will be diverted to lactate, thereby assuring regeneration of NAD^+ ^from NADH. This will enable glycolysis, and the accompanying ATP production to proceed.

The blood lactate concentration reflects a balance between production and uptake of lactate in tissues, and is normally between 0.5-1.8 mM. Lactate is formed by reduction of pyruvate, and is metabolized by oxidation to pyruvate in the reaction catalyzed by the cytosolic NAD-dependent lactate dehydrogenase (Figure 1). The metabolic fate of pyruvate is mainly mitochondrial oxidation to carbon dioxide and water with accompanying energy production in the respiratory chain. The latter sequence of reactions are oxygen requiring, and with insufficient oxygen supply, or if pyruvate production for other reasons exceeds the capacity of oxidative metabolism, pyruvate will be diverted to lactate. This assures regeneration of NAD^+ ^from NADH, which will enable glycolysis, and the accompanying ATP production to proceed. Due to the central role of the NAD-redox state for lactate production and metabolism, any metabolic condition giving rise to a steady-state increase in the cytosolic NADH/NAD^+ ^ratio, will cause an increased net lactate production. This applies not only to conditions of hypoxia/anoxia in all tissues, but is also observed e.g. during extensive muscular work, and during alcohol metabolism by the liver. Lactate is released from tissues accompanied by a proton, and because lactic acid is fully dissociated at pH above approximately 6, excessive lactate production may thus give rise to lactic acidosis. The uptake of lactate from plasma takes place predominantly in liver and heart, where lactate will be used as an energy producing substrate or, in case of the liver, as a precursor for glucose formation.

## Methods

### Eligibility criteria

Due to the lack of studies of high quality, it was a priori decided to allow the inclusion of all study types. Randomized, controlled trials were prioritized, but also non-randomized trials, cohort studies, and case-control studies were considered. Case studies, studies with fewer than 40 patients, and abstracts where full text articles were not available were excluded. This review was restricted to English language publications. Study characteristics were defined according to PICO:

Participants: patients in the acute setting, i.e. patients assessed pre-hospitally, in the trauma centre, ED, or ICU.

Intervention/exposure: elevated lactate levels in venous, arterial or capillary blood.

Comparisons: patients with lactate within the normal range. Outcome: in-hospital mortality.

Inclusion and exclusion criteria are listed in Table 1.

**Table 1:** Inclusion and exclusion criteria

Inclusion criteria	Exclusion criteria
∙ Blood lactate assessment in the acute setting: pre-hospital, emergency department, trauma centre, or intensive care unit	∙ Abstract only
∙ Any age	∙ Case study
∙ Single or serial lactate measured in arterial, venous, or capillary blood	∙ Fewer than 40 patients included
∙ Outcome measure: in-hospital mortality	∙ Based on specific types of post-operative patients
∙ Written in English	
∙ Human studies	

### Data sources

Studies were identified by a systematic search using PubMed, Cochrane Central Register of Controlled Trials, Cochrane Database of Systematic Reviews, and CINAHL up to April 2011. Reference lists of relevant peer-reviewed literature were hand searched to identify any appropriate articles that may have been missed by the electronic search.

### Search strategy

We searched PUBMED systematically using the Medical Subject Headings (MeSH),

("Lactic Acid/blood"[Mesh] OR "Lactic Acid/diagnostic use"[Mesh] OR "Acidosis, Lactic/blood"[Mesh] OR "Acidosis, Lactic/classification"[Mesh] OR "Acidosis, Lactic/complications"[Mesh] OR "Acidosis, Lactic/diagnosis"[Mesh] OR "Acidosis, Lactic/drug therapy"[Mesh] OR "Acidosis, Lactic/etiology"[Mesh] OR "Acidosis, Lactic/metabolism"[Mesh] OR "Acidosis, Lactic/mortality"[Mesh] OR "Acidosis, Lactic/physiopathology"[Mesh] OR "Acidosis, Lactic/prevention and control"[Mesh] OR "Acidosis, Lactic/surgery"[Mesh]) AND ("triage"[Mesh] OR "Injury Severity Score"[Mesh] OR "Mortality"[Mesh] OR "Blood Component Transfusion"[Mesh] OR "Emergency medical services"[Mesh] OR "Emergency Service, Hospital"[Mesh] OR "Sepsis"[Mesh] OR "Point-of-care systems"[Mesh] OR "Intensive care units"[Mesh] OR "Intensive care"[Mesh] OR "Length of stay"[Mesh] OR "Early diagnosis"[Mesh] OR "Critical illness"[Mesh] OR "Shock"[Mesh] OR "Treatment Outcome"[Mesh] OR "Veins"[Mesh] OR "Arteries"[Mesh] OR "Capillaries"[Mesh])

### Study selection and data extraction

Two experts independently screened the selected studies for inclusion. Studies were included only if both experts considered the study as relevant for answering the study objectives.

Information was extracted from each included study on (1) characteristics of participants; (2) characteristics of blood lactate assessment; (3) lactate cut off value; (4) type of outcome measure; (5) statistics supporting the main findings of the study. To assess the quality of the included studies, methods validated for internal validity, precision, and applicability (external validity) were applied [9]. The methodological quality and clinical relevance of each study was graded as high, moderate, or low. The review was presented according to 'Preferred Reporting Items for Systematic Reviews and Meta-Analysis: 'The PRISMA statement' [10].

## Results

The initial search identified 1.677 abstracts, which were evaluated for relevance. 66 articles were considered potentially relevant and evaluated in full text. Ultimately 33 articles were selected.

**Figure 2:**
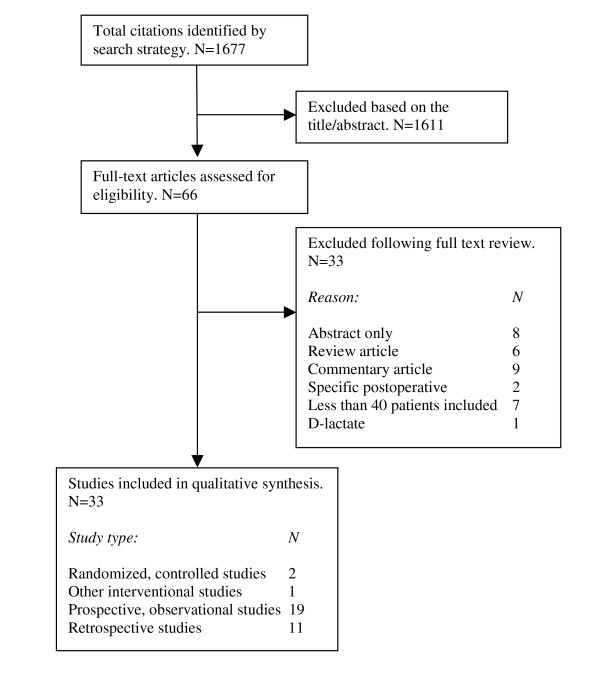
**Flow diagram**. Results of literature search and selection process.

The flow diagram in Figure 2 illustrates the study selection.

The articles that were ultimately selected were divided in 2 groups; each group referring to one of the two research questions. The groups were:

1) Studies investigating single or serial blood lactate assessment (Table 2).

2) Studies comparing arterial, venous and capillary lactate (Table 3).

**Table 2:** Studies investigating predictive value of single or serial blood lactate assessment.

Author, year, reference	Study design, study quality*	N	Patient characteristics	Lactate characteristics	Outcome measure	Cut off [mM]	Main findings
Shapiro et al., 2005 [2]	Prospective, observational **Moderate** Acceptable external validity. Acceptable internal validity. Sample size of uncertain adequacy.	1278	ED patients with infection-related diagnosis (≥ 18 years).	Venous lactate at admission to ED.	3-day and 28-day in-hospital mortality.	0-2.5; 2.5-4.0; > 4.0	Mortality rate increased with lactate levels: 0-2.5 mM: 4.9%; 2.5-4.0 mM: 9%; > 4.0 mM: 28%. Area under ROC curve for 3-day mortality was 0.80; for 28-day mortality it was 0.67. For 28-day mortality lactate between 2.5-4.0 mM had sens. 59% and spec. 71%. Lactate > 4.0 mM had sens. 36% and spec. 92%. For 3-day mortality lactate between 2.5-4.0 mM had sens. 76% and spec. 71%. Lactate > 4.0 mM had sens. 55% and spec. 91%.

Callaway et al., 2009 [3]	Retrospective **Moderate** Acceptable external validity. Acceptable internal validity. Sample size of uncertain adequacy.	588	Normotensive blunt trauma patients (≥ 65 years).	Venous lactate at admission to ED.	In-hospital mortality.	2.5	Compared to patients with admission lactate < 2.5 mM, lactate > 4.0 mM had OR 4.2 (2.4-7.5) for death. Area under ROC curve was 0.60.

Howell et al., 2007 [4]	Prospective, observational **Moderate** Acceptable external validity. Good internal validity. Sample size of uncertain adequacy.	1287	ED patients with infection-related diagnosis.	Venous lactate at admission to ED.	28-day in-hospital mortality.	2.5-4.0; > 4.0	Admission lactate predicted 28-day mortality independently of blood pressure (p < 0.0001). Compared to lactate < 2.5 mM, lactate between 2.5-4.0 mM had OR 2.2 (1.1-4.2). Lactate > 4.0 mM had OR 7.1 (3.6-13.9). Area under ROC curve was 0.87.

Khosravani et al., 2009 [11]	Retrospective **Moderate** Acceptable external validity. Acceptable internal validity. Sample size of uncertain adequacy.	9036	Intensive care patients, unspecified (≥ 18 years).	Arterial or venous lactate at admission to ICU.	ICU mortality.	2.0	Lactate was an independent predictor of mortality: 2-5 mM: OR 1.94 (1.62-2.32); 5-10 mM: OR 3.38 (2.64-4.33); 10-15 mM: OR 4.41 (2.99-6.5); 15-20 mM: OR 7.58 (3.93-14.6); 20-max: OR 10.89 (4.85-24.48). All compared to control group with lactate < 2 mM

Nichol et al., 2010 [12]	Retrospective **Moderate** Acceptable external validity. Good internal validity. Sample size of uncertain adequacy.	7155	Intensive care patients, unspecified.	Arterial lactate at admission to ICU. Serial: interval unspecified.	In-hospital mortality.	2.0	Compared to lactate < 0.75 mM, admission lactate > 2.0 mM had OR for mortality at 2.1 (1.3-3.5, p = 0.01). Sustained lactate between 0.75-1.0 mM had OR = 2.0 (p < 0.0001). Sustained lactate > 2.0 mM had OR = 3.7 (1.9-7.0, p < 0.0001).

Smith et al., 2001[13]	Prospective, observational **Moderate** Acceptable external validity. Acceptable internal validity. Sample size of uncertain adequacy.	148	Intensive care patients, unspecified.	Arterial lactate at admission to ICU. Serial: 24 hours later.	28-day in-hospital mortality.	1.5	Admission lactate > 1.5 mM was associated with 28-day mortality (p < 0.0001). Area under ROC curve = 0.78. Patients with lactate > 1.0 mM at 24 hours have significantly higher mortality (p = 0.0001).

Suistomaa et al., 2000 [14]	Prospective, observational **Moderate** Acceptable external validity. Acceptable internal validity. Sample size of uncertain adequacy.	98	Intensive care patients, unspecified.	Arterial lactate at admission to ICU. Serial: every 2 hours the first 24 hours.	In-hospital mortality.	2.0	Median peak lactate for non-survivors was 5.3 mM (IQR, 1.9-7.5) vs. 1.9 mM (IQR, 1.3-2.9) for survivors, p = 0.003. Hyperlactatemia at admission to ICU was associated with higher mortality than hyperlactatemia that developed after admission (29.0% vs. 5.9%, p = 0.003). Persistent hyperlactatemia (> 6 hours) was associated with higher mortality than transient hyperlactatemia (36.8% vs. 0%, p = 0.008).

Hatherill et al., 2000 [15]	Prospective, observational **Low** Acceptable external validity. Uncertain internal validity. Probably underpowered study.	50	Children at ICU with shock, and initial hyperlactatemia.	Arterial lactate at admission to ICU. Serial: at 24 hours, and additionally at unspecified intervals.	ICU mortality.	2.0	The area under the ROC curve for all values of lactate > 2 mM on admission was 0.59. Persistent hyperlactataemia > 2 mM after 24 hours was associated with 93% mortality, as compared to 30% in those children whose lactate level had normalised. Persistent hyperlactataemia at 24 hours identified mortality with a likelihood ratio of 7, sens. 78%, and spec. 89%. The area under the ROC curve for lactate > 2 mM at 24 h after admission was 0.86.

Cerovic et al., 2003 [16]	Prospective, observational **Low** Acceptable external validity. Good internal validity. Probably underpowered study.	94	Seriously injured patients defined as ISS ≥ 16 who survived ≥ 12 hours.	Arterial lactate at admission to ICU. Serial: every 12 hours during the first 48 hours after admission.	In-hospital mortality.	2.0	Admission lactate was not a significant predictor for mortality. Survivors exhibited a progressive decline in lactate levels, while lactate in the non-survivors remained broadly unchanged from the 12th hour after the first sampling.

del Portal et al., 2010 [17]	Retrospective **Moderate** Acceptable external validity. Good internal validity. Sample size of uncertain adequacy.	1442	ED patients (≥ 65 years).	Lactate at admission to ED.	30-day and 60-day in-hospital mortality.	2.0	Admission lactate were linearly associated with mortality (RR = 1.9 to 3.9) depending on lactate levels (p < 0.01).

Jansen et al., 2008 [18]	Prospective, observational **Moderate** Acceptable external validity. Acceptable internal validity. Sample size of uncertain adequacy.	124	Patients who required urgent ambulance dispatching with systolic blood pressure < 100 mmHg, or respiration rate < 10, or > 29, or GCS < 14.	Pre-hospital venous or capillary lactate arrival on the site of injury and at admission to the hospital.	In-hospital mortality.	3.5	Mortality was significantly higher in patients with lactate ≥ 3.5 mM at the site of injury (41% vs. 12%; p < 0.001) or at admission to the hospital (47% vs.15%; p < 0.001). Lactate, on average, increased 0.1 mM in non-survivors, whereas in survivors, it decreased 0.6 mM (p = 0.044) Pre-hospital lactate had better prognostic value than vital signs alone.

Kaplan et al., 2004 [19]	Retrospective **Moderate** Acceptable external validity. Acceptable internal validity. Sample size of uncertain adequacy.	282	Trauma patients with vascular injury (torso or extremity).	Arterial lactate at admission to trauma center.	28-day in-hospital mortality.		Admission lactate could discriminate survivors from non-survivors (3.6 mM vs. 11.1 mM, p < 0.001).

Pal et al., 2006 [20]	Retrospective **Moderate** Acceptable external validity. Acceptable internal validity. Sample size of uncertain adequacy.	5995	Trauma patients, unspecified.	Arterial lactate at admission to trauma center.	In-hospital mortality.	2.0	Survivors had 3.0 mM, and non-survivors had 5.2 mM (p < 0.0001). Sens. and spec. of an elevated lactate was 85% and 38%, respectively. Area under ROC curve was 0.72. PPV was 4%.

Vandromme et al., 2010 [21]	Retrospective **Moderate** Acceptable external validity. Acceptable internal validity. Sample size of uncertain adequacy.	2413	Trauma patients with systolic blood pressure between 90 and 110 mmHg.	Capillary or venous lactate at admission to hospital.	Need for ≥ 6 units packed red blood cells within 24 hours. In-hospital mortality.	2.5	Admission lactate was a better predictor for mortality and need for blood transfusion than systolic blood pressure (p < 0.0001). Lactate had area under ROC curve = 0.76 and systolic blood pressure had area under ROC curve = 0.61, p < 0.0001.

Arnold et al., 2009 [22]	Retrospective **Low** Acceptable external validity. Good internal validity. Probably underpowered study.	166	ED patients diagnosed with severe sepsis. (> 17 years)	Venous lactate at admission to ED. Serial: interval unspecified.	In-hospital mortality	4.0	The mean initial lactate for survivors was 4.3 mM (SD = 2.6), while non-survivors had 4.7 mM (SD = 2.8), p = 0.41. The mean serial lactate for survivors was 2.2 mM (SD = 1.6), while non-survivors had 3.6 mM (SD = 2.8), p < 0.001.

Guyette et al., 2011 [23]	Retrospective **Moderate** Acceptable external validity. Good internal validity. Sample size of uncertain adequacy.	1168	Trauma patients transported by air. (≥ 18 years)	Pre-hospital venous or capillary lactate was measured	In-hospital mortality	2.0	Pre-hospital lactate was median 3.8 mM (IQR, 2.8-6.1) in those who died and median 2.3 mM (IQR, 1.3-3.4) in those who survived to discharge, p < 0.0001.

Trzeciak et al. 2007 [24]	Prospective, observational **Low** Acceptable external validity. Uncertain internal validity. Sample size of uncertain adequacy.	1177	ED patients with diagnosis of sepsis or infection. (> 18 years).	Venous lactate at admission to ED.	In-hospital mortality and death within 3 days from measurement	4.0	Compared to baseline 0.0-2.0 mM, patients with lactate ≥ 4.0 mM had OR 6.1(3.7-10.5) for dying within 3 days from lactate measurement. Sens. 35%. Spec. 92%. Area under ROC curve 0.63. Equivalently lactate ≥ 4.0 mM had OR 3.0(2.0-4.6) for in-hospital death. Sens. 19%. Spec. 93%. Area under ROC curve was 0.56.

Kaplan et al., 2008 [25]	Retrospective **Low** Acceptable external validity. Good internal validity. Probably underpowered study.	78	Patients with blunt, or penetrating trauma requiring intensive care	Arterial lactate at admission to ED.	28-day in-hospital mortality.	2.2	Admission lactate could not discriminate survivors from non-survivors (2.3 mM vs. 2.9 m, p = 0.24). Area under ROC curve was 0.6.

Van Beest et al., 2009 [27]	Prospective, observational **Moderate** Acceptable external validity. Acceptable internal validity. Sample size of uncertain adequacy.	135	Patients with at least 2 symptoms of shock. (≥ 18 years)	Pre-hospital capillary or venous lactate.	Length of stay, and in-hospital mortality.	4.0	Hyperlactamic patients had significantly higher mortality (12.2% vs. 44.3%, p = 0.002), longer LOS at ICU (p = 0.03), and LOS in hospital (p = 0.04). Area under ROC curve was 0.775. Lactate > 3.2 mM was the optimal cut off with sens. 75% and spec. 72%.

Nguyen et al., 2004 [28]	Prospective, observational **Moderate** Acceptable external validity. Good internal validity. Sample size of uncertain adequacy.	111	Patients with severe sepsis or septic shock. (> 18 years).	Arterial lactate at admission to ED. Serial: at 6 hours.	60-day in-hospital mortality.		** Lactate clearance less than 10% in 6 hours was associated with a higher 60-day mortality, than lactate clearance more than 10% (p = 0.007). Sens. 44.7% and spec. 84.4%.

Claridge et al., 2000 [29]	Prospective, observational **Moderate** Acceptable external validity. Good internal validity. Sample size of uncertain adequacy.	381	Trauma patients requiring intensive care.	Lactate at admission to ED. Serial: every 4-6 hours.	In-hospital mortality.	2.4	** Patients with sustained hyperlactatemia (> 12 hours) had higher risk of infection (pneumonia etc.) than controls (69.8% vs. 40%, p < 0.001). The mortality rate for patients who developed infection was 7.9% vs. 1.9% (p < 0.05).

Jansen et al., 2009 [30]	Prospective, observational **Low** Acceptable external validity. Good internal validity. Probably underpowered study.	394	Intensive care patients with sepsis, hemorrhage, or other conditions of low oxygen transport.	Arterial lactate at admission to ICU. Serial: 12 and 24 hours after admission.	In-hospital mortality.	2.5	** Reduction of lactate within 24 hours was associated with significantly lower mortality in the septic group (p = 0.003), but not in the other groups, (p = 0.42).

Kliegel et al., 2004 [31]	Retrospective **Moderate** Acceptable external validity. Acceptable internal validity. Sample size of uncertain adequacy.	394	Patients resuscitated after cardiac arrest who survived > 48 hours.	Arterial lactate. First sample at admission to ED. Serial: every 4-8 hours.	In- hospital mortality.	2.0	On admission survivors had 7.8 mM (IQR, 5.4-10.8) and non-survivors had 9 mM (IQR, 6.5-11.9), p < 0.01. Lactate > 2 mM after 48 hours predicted mortality with a spec. of 86%, and poor neurologic outcome with a spec. of 87%. Sens. for both were 31%.

Jansen et al., 2010 [32]	Randomised, controlled trial **High** Acceptable external validity. Good internal validity. Premeditated and sufficient study size.	348	Intensive care patients, unspecified. (≥ 18 years).	Arterial lactate at admission to ICU. (venous and capillary lactate was also allowed). Serial: every 2 hours.	In-hospital mortality.	3.0	** Hazard ratio in the intervention group was 0.61 (0.43-0.87, p = 0.006). Reduction of lactate below 2.0 mM was not associated with better outcome.

Jones et al., 2010 [33]	Randomised, controlled trial **High** Acceptable external validity. Good internal validity. Premeditated and sufficient study size.	300	Patients with severe sepsis and hypoperfusion or septic shock. (≥ 17 years).	Venous lactate at admission to ICU. Serial: every hour.	In-hospital mortality.	2.0	** No difference in mortality was observed in patients treated by a protocol aiming at normalizing blood lactate compared to normalizing SvO2.

Blow et al., 1999 [43]	Prospective, intervention **Low** Acceptable external validity. Uncertain internal validity. Probably underpowered study.	79	Hemo-dynamic stable trauma patients with ISS ≥ 20 and survival > 24 hours.	Lactate at admission to trauma centre. Serial: interval unspecified.	In-hospital mortality.	2.5	** Persistent hyperlactatemia after 24 hours was associated with increased mortality (p < 0.05).

Lee et al., 2008 [44]	Prospective, observational **Low** Acceptable external validity. Good internal validity. Probably underpowered study.	126	Patients with severe sepsis, or septic shock. (≥ 20 years).	Arterial lactate at admission to ED. Serial: 4 hours later.	In-hospital mortality.	2.0	** No significant difference in mortality was found between patients with elevated lactate compared to normal lactate, as long as pH was within normal limits.

Numbers in brackets are 95% confidence interval unless specified otherwise. *quality rated by using methods validated for internal validity, precision, and applicability (external validity) [9]. **Predictive value of admission lactate in relation to mortality not commented. ED = emergency department; ROC = receiver operating characteristic; sens. = sensitivity; spec. = specificity; OR = odds ratio; ICU = intensive care unit; IQR = interquartile range; ISS = injury severity score; RR = relative risk; GCS = Glascow coma scale; PPV = positive predictive value; SD = standard deviation; LOS = length of stay; SvO_2 _= central venous oxygen saturation.

**Table 3:** Studies with comparison of arterial, venous, and capillary lactate.

Author, year, reference	Study design, study quality*	N	Patient characteristics	Lactate characteristics	Outcome measure	Cut off [mM]	Main findings
Lavery et al., 2000 [26]	Prospective, observational **Moderate** Acceptable external validity. Acceptable internal validity. Sample size of uncertain adequacy.	231	Trauma patients, unselected.	Arterial and peripheral venous lactate within 10 min after admission at trauma center.	Comparison of arterial and venous lactate.	2.0	No significant difference between arterial and venous lactate was found. The correlations were: a.femoralis-v.femoralis, r = 0.95; a.femoralis-peripheral vein, r = 0.935; a.radialis-peripheral vein, r = 0.988. No significant difference was found for peripheral venous lactate drawn with or without the use of tourniquet. For LOS > 2 days, lactate > 2.0 mM had sens. 68%, spec. 41%, PPV 71%, and NPV 48%. For in-hospital mortality, lactate > 2.0 mM had sens. 95%, spec. 43%, PPV 16%, and NPV 99%.

Boldt et al., 2001 [34]	Prospective, observational **Moderate** Acceptable external validity. Acceptable internal validity. Sample size of uncertain adequacy.	40	Surgical intensive care patients, unselected.	Arterial and capillary lactate at inclusion day, after 8 hours, and once per day the following three days.	Compares POCT and laboratory lactate. Comparison of arterial and capillary lactate.	2.0	Correlation between POCT and the hospital laboratory for measuring lactate in arterial blood was r^2 ^= 0.97. Bland-Altman showed bias 0.15 mM with POCT tending to be lower. Correlation between capillary and arterial blood was r^2 ^= 0.80. Bland-Altman showed bias 0.59 mM with capillary lactate tending to be higher.

Perez et al., 2008 [35]	Prospective, observational **Moderate** Acceptable external validity. Acceptable internal validity. Sample size of uncertain adequacy.	120	Patients in antiretroviral therapy with hyperlactatemia.	Venous lactate.	Compares POCT and reference-instrument.	2.2	Correlation between mean venous lactate measured with POCT and reference instrument (2.89 mM vs. 2.78 mM), r = 0.63. The POCT instrument had a sensitivity of 95.9%, and a specificity of 63.8% for hyperlactatemia. PPV was 80.5%. NPV was 90.9%. Agreement was analysed by Bland-Altman plot. Bias was 0.113 mM (-2.103-2.329) with POCT tending to show higher values.

Shapiro et al., 2010 [36]	Prospective, observational **Moderate** Acceptable external validity. Acceptable internal validity. Sample size of uncertain adequacy.	699	Patients with suspected infection (pneumonia etc.). (≥ 18 years).	Venous lactate in the emergency department. Convenience sample.	Comparison of POCT venous lactate and laboratory venous control. In-hospital mortality.		Correlation between venous lactate measured by POCT and laboratory was 0.97. POCT lactate had area under ROC curve = 0.72 for mortality. Bland-Altman plot showed that POCT lactate was, on average, 0.32 mM (-0.35-0.98) lower than laboratory lactate, with agreement kappa = 0.97.

Gallagher et al., 1997 [37]	Prospective, observational **Moderate** Acceptable external validity. Acceptable internal validity. Sample size of uncertain adequacy.	69	Emergency department patients (≥ 18 years).	Arterial and peripheral venous lactate. Convenience sample.	Comparison of arterial and peripheral venous lactate.	1.6	Correlation between arterial and peripheral venous lactate was r^2 ^= 0.89. There was no correlation between the arterio-venous difference and tourniquet time (r^2 ^= 0.02). Peripheral venous lactate had a sensitivity of 94%, and a specificity of 57% for hyperlactatemia. Bias was 0.22 mM (0.04-0.41) with venous lactate tending to be higher.

Younger et al., 1996 [38]	Prospective, observational **Moderate** Acceptable external validity. Acceptable internal validity. Sample size of uncertain adequacy.	48	Emergency department adult patients. (≥ 17 years).	Arterial and peripheral venous lactate.	Comparison of arterial and peripheral venous lactate.	1.6	Peripheral venous lactate > 1.6 mM has sensitivity of 100%, and specificity of 86% for hyperlactatemia. The correlation between arterial and venous lactate was r^2 ^= 0.71 (p < 0.001). Bland-Altman plot showed bias 0.18 mM (0.012-0.372) with venous lactate tending to be higher.

Numbers in brackets are 95% confidence interval unless specified otherwise. *quality rated by using methods validated for internal validity, precision, and applicability (external validity) [9]. LOS = length of stay; sens. = sensitivity; spec. = specificity; PPV = positive predictive value; NPV = negative predictive value; POCT = point-of-care testing; ROC = receiver operating characteristic.

### Single blood lactate assessment

The predictive value of a single elevated blood lactate on mortality is demonstrated in a number of studies in patients admitted to ICU [11-16]. Four of these studies were of moderate quality and two were of low quality. Admission lactate ≥ 2 mM was a significant independent predictor of mortality in adult patients admitted to ICU in a large retrospective study by Khosravani et al. [11]. Odds Ratio (OR) for mortality increased form 1.94 to 10.89 dependent on the level of hyperlactatemia, compared to patient with admission lactate < 2 mM. Studies by Nichol et al. and Smith et al. also demonstrated a highly significant effect of an elevated admission lactate above 2.0 mM respectively 1.5 mM on mortality in unselected ICU patients [12,13]. In patients admitted acutely to ED or trauma centre several studies have demonstrated a significant effect of elevated admission lactate on in-hospital mortality [2-4,17-26]. Ten of these studies were of moderate quality, and three were of low quality. The admission lactate in this group of patients was either assessed pre-hospitally, by admission to the ED, or the ICU. The cut-off value was chosen between 2.0 and 4.0 mM. Two minor studies were not able to demonstrate a significant difference between survivors and non-survivors based on a single elevated lactate measurement [22,25]. In addition to in-hospital mortality as the primary outcome measure, some studies also demonstrated a significant effect on secondary outcome measures; for instance length of hospital stay [26,27] and the need for blood transfusion within the first 24 hours [21]. Several of the studies express a dose-response relationship between lactate levels and mortality [2,4,11,12,17].

### Serial blood lactate assessment

Sustained hyperlactatemia among ICU patients, demonstrated by serial measurements, has shown to be predictive for in-hospital mortality [12-16,28-30]. Five of these studies were of moderate quality and three were of low quality. In these studies, high lactate levels in serial measurements and prolonged time to normalize lactate, predicted a higher mortality rate. Most studies used 2.0 mM as cut off point. In a large study by Nichol et al., however, sustained lactate levels as low as 0.75-1.0 mM were associated with doubling of the risk of adverse outcome (OR = 2.0, p < 0.0001) [12]. In 2004 Kliegel et al. examined patients who were resuscitated from cardiac arrest and survived at least 48 hours [31]. They concluded that sustained hyperlactatemia (> 2.0 mM after 48 hours) was predictive for mortality as well as poor neurological outcome. Only one single study has evaluated the value of serial lactate monitoring pre-hospitally [18]. In this study, Jansen et al. assessed lactate at the site of injury and at arrival to the hospital, and found that mortality was significantly higher in patients with lactate > 3.5 mM at the site of injury (41% vs. 12%, p < 0.001), or at admission to the hospital (47% vs. 15%; p < 0.001) compared to patients with lactate levels < 3.5 mM. Pre-hospital lactate had better prognostic value than vital signs such as blood pressure alone. The effect of lactate guided treatment was studied in a randomized controlled study of ICU patients [32]. The administration of erythrocytes, dobutamine, and fluid substitution was administrated following an algorithm targeting a SvO_2 _at 70%. In the intervention group further therapy with vasodilators was started if lactate clearance was less than 20% in 2 hours. When adjusted for predefined risk factors, in-hospital mortality was significantly lower in the intervention group (hazard ratio 0.61; 95% CI 0.43-0.87, p = 0.006). No further reduction in mortality was observed when lactate levels were reduced to less than 2 mM. In a randomized controlled study by Jones et al., septic patients and other patients with hypoperfusion were treated by two different resuscitation protocols, one guided by SvO_2 _and the other guided by lactate levels [33]. There was no significant difference in mortality between the two groups.

### Comparison of arterial, venous, and capillary lactate

Point-of-care testing has enabled monitoring of lactate levels close to the patients in the general ward, in the ambulance, or in the ED. Six studies investigating arterial, venous, and capillary lactate were included. All studies were of moderate quality. High accuracy was demonstrated in a number of studies, when lactate was analyzed using POCT compared to laboratory results in the same type of blood (venous, arterial, or capillary) [34-36]. The correlation between arterial and capillary values of lactate was evaluated in 2001 by Boldt et al., who showed a moderate correlation between lactate measured in capillary blood compared to arterial values (r^2 ^= 0,80) in 40 intensive care patients [34]. They recommended that capillary lactate should not substitute arterial lactate in critically ill patients due to the risk of overestimating lactate levels. The correlation between arterial and peripheral venous lactate has been investigated by Lavery et al., who found no significant difference between arterial and venous levels of lactate, the highest correlation being between the radial artery and a peripheral vein (r = 0.988) [26]. In a study with patients admitted to ED, Gallagher et al. found a correlation at r^2 ^= 0.89 between arterial and venous lactate [37]. Venous lactate tended to be overestimated (bias 0.22 mM, 95% CI (0.04-0.41)). Younger et al. found a moderate correlation between arterial and venous lactate (r^2 ^= 0.71) in patients admitted to ED, also with tendency of venous lactate being overestimated (bias 0.18 mM, 95% CI (0.012-0.372)) [38]. The sensitivity and specificity of peripheral venous lactate for hyperlactatemia were found to be 94% and 57% respectively in the study by Gallagher et al., while sensitivity was 100% and specificity was 86% in the study by Younger et al. [37,38]. No difference was found in peripheral venous lactate level when comparing sampling with or without the use of tourniquet [26,37].

## Discussion

### Single versus serial lactate monitoring

Most studies reviewed here support single lactate measurements, assessed at admission to the hospital, as being useful in terms of predicting adverse outcome. The predictive value of blood lactate is further supported by the fact that several studies demonstrate a dose-response relationship; the higher the lactate levels, the higher the mortality rates. The predictive value of a single lactate assessed at admission is, however, controversial, as several studies were not able to show a significant predictive value of admission lactate [16,22,25]. Furthermore there is great heterogeneity between studies when defining, at what stage an admission lactate should be taken; some studies choosing the first patient contact, others when the patient is admitted to ICU. In the population admitted to ICU, close monitoring of all patients are mandatory, and it seems reasonable to perform serial lactate measurement on all patients. However in trauma patients or patients admitted to ED, a decision based on a single lactate measurement could be useful in terms of deciding the level of observation and treatment, and to select patients for serial lactate monitoring. Sustained hyperlactatemia has been shown to be predictive for adverse outcome in a number of studies, and therefore serial lactate measurements might be a useful approach to monitor the critically ill patient. Another advantage by using serial measurements is that patients having a temporary and non-pathologically elevated lactate, for instance as a result of a high adrenaline level [39], or alcohol intake [40], are excluded from the population. There is no agreement about the optimal time interval between serial lactate sampling. Jansen et al. aimed to reduce lactate by 20% per 2 hours, and managed to reduce mortality significantly in the intervention group [32]. Therefore, based on the reviewed articles, an interval between two and six hours seems reasonable to be able to follow the trend.

### Lactate cut off point

This study was not designed to specify the appropriate lactate cut off values. Setting the cut off point for lactate in order to predict patient outcome, is a question of getting the optimal relationship between sensitivity and specificity. This is most easily visualized by the receiver operating characteristics (ROC) [41]. A test with high sensitivity carries the risk of many false positive and hence over-triage. On the other hand, if the test is used for screening purposes, or as a part of multifactorial risk assessment, it might be desirable with a certain level of over-triage. The higher the cut off point, the better predictive value of a positive test, and the greater risk for getting false negative results. Several of the studies demonstrating a significant predictive value of single lactate, assessed at admission to hospital, had a cut off point at 2.5 mM [2,4,21]. Studies which used a higher cut off point, also found significant predictive value of admission lactate [18,24]. When 2.0 mM was used as cut off point, the predictive value of admission lactate became more controversial. This indicates that the cut off point for lactate, assessed at admission to hospital, probably should lie in the interval 2.0-2.5 mM. The cut off point for sustained hyperlactatemia are for several studies 2.0 mM [12,14-16,31]. A few studies even found significantly increased mortality for a sustained lactate as low as 0.75-1.0 mM [12,13]. More studies are needed to support this very low cut off point; it will inevitably lead to the risk of over-triage. It is possible however, that the limit for serial lactate should be considered lower than for admission lactate.

### Lactate measured in capillary, arterial, or venous blood

The golden standard in assessing lactate levels is in arterial blood. In a previous review, the authors concluded that most studies found a satisfactory agreement between the capillary, peripheral venous, and arterial lactate [1]. The studies available, however, are all quite small, and they present conflicting conclusions in relation to whether the capillary lactate could replace the arterial, due to tendency of a higher lactate result in capillary blood. Peripheral venous lactate demonstrates a good correlation to the arterial lactate, as well as a very high sensitivity and acceptable specificity for hyperlactatemia. Bland-Altman plots show bias between 0.18-0.22 mM, with the tendency of venous lactate being higher than arterial lactate [37,38]. Setting the same cut-off for arterial and venous lactate levels will lead to more false positive results when using venous lactate, but makes lactate in peripheral blood a reasonable screening test, because all patients with hyperlactatemia are found. An arterial puncture requires training of the personnel, can be time consuming, expensive, and subject to pain and inconvenience for the patient [26,34]. A venous or capillary lactate would increase the accessibility of the test, because the sample could be drawn together with other venous blood samples at admission to hospital, or in the ambulance. Furthermore a venous or capillary sample carries minimal risk and inconvenience for the patient, and minimal training of the personnel. The use of capillary lactate carries the risk of over-triage, unless the cut off point is set higher than for arterial lactate.

### Strengths and limitations

The strength of this review is due to the systematic search and inclusion of studies. The quality of the studies reviewed is, however, not optimal. The majority of the studies presented here are of moderate or low quality, and suffers from a sample size of uncertain adequacy; some of the studies are quite small and probably underpowered. Furthermore convenience samples are used in the studies comparing lactate levels in capillary, arterial, and venous blood with the associated risk of getting selection bias. The lactate taken at admission is not clearly defined; the blood sample is taken in the ED, the ICU, or pre-hospitally depending on the study design. In addition, the population is heterogeneous and in different phases of the acute process. We find, that an attempt to evaluate the strength of the recommendation for monitoring lactate, for instance using the internationally accepted GRADE system, is not possible due to the heterogeneity between studies [42]. This is in accordance with the conclusion of Jansen et al, in a recent review discussing the use of lactate monitoring [1]

### Future research

The lactate cut off values should be confirmed by larger, prospective studies. These should be followed by interventional studies to determine if therapy directed towards a reduction in lactate levels improve patient outcome compared to a control group. Lactate level is a surrogate parameter for the insufficiency of the circulation. Therefore all interventions aiming at improving the circulation (fluids, inotropes etc.) will have an effect on lactate levels. It is probably not the elevated lactate per se, but the underlying condition that increases the risk for adverse outcome. This needs to be further investigated. Concerning the discussion about the use of capillary and peripheral lactate, a study with a larger number of patients is wanted. The study should aim to take arterial, peripheral venous, and capillary lactate within a short time relative to each other, and preferably serial measurements over time. It would be beneficial to include a subpopulation of patients with hypotension, since peripheral constriction, triggered by increased sympathetic activity, could lead to artificially high lactate values when measured on capillary blood.

## Conclusions

Blood lactate monitoring is useful for risk assessment in patients admitted acutely to hospital, and especially the trend in serial lactate monitoring is valuable in predicting in-hospital mortality. All patients with a lactate at admission above 2.5 mM should be closely monitored for signs of deterioration, and patients with even lower lactate levels should be considered for serial lactate monitoring. Peripheral venous blood lactate is highly correlated with arterial blood lactate, and venous sampling should therefore be encouraged, as the risk and inconvenience for this procedure is minimal for the patient. The relevance of lactate guided therapy has to be supported by more studies.

## List of abbreviations

CI: confidence interval, ED: emergency department, GCS: Glasgow coma scale, ICU: intensive care unit, IQR: interquartile range, ISS: injury severity score, LOS: length of stay, NPV: negative predictive value, OR: odds ratio, POCT: point-of-care testing, PPV: positive predictive value, RR: relative risk, ROC: receiver operating characteristic, Sens.: sensitivity, Spec.: specificity, SvO_2 _: central venous oxygen saturation.

## Competing interests

The authors declare that they have no competing interests.

## Authors' contributions

All authors contributed to study concept and design and acquisition, analysis and interpretation of the data. Finally all authors read and approved the submitted manuscript.
